# Phytogenic feed additives as alternatives to antibiotics in poultry production: A review

**DOI:** 10.14202/vetworld.2025.141-154

**Published:** 2025-01-22

**Authors:** Noor Aminullah, Allauddin Mostamand, Ahmadullah Zahir, Obaidullah Mahaq, Mohammad Naeem Azizi

**Affiliations:** 1 Department of Pri-Clinic, Faculty of Veterinary Science, Afghanistan National Agricultural Sciences and Technology University, Kandahar 3801, Afghanistan; 2 Department of Animal Husbandry, Faculty of Animal Science, Afghanistan National Agricultural Sciences and Technology University, Kandahar 3801, Afghanistan; 3 Department of Food Science and Technology, Faculty of Veterinary Science, Afghanistan National Agricultural Sciences and Technology University, Kandahar 3801, Afghanistan; 4 Department of Animal Nutrition and Production, Faculty of Agriculture, Afghan International Islamic University, Kabul 1004, Afghanistan

**Keywords:** alternatives, antibiotics, phytogenic, poultry production

## Abstract

The overuse of antimicrobials in food-producing animals, particularly poultry, has led to growing concerns about multidrug microbial resistance, posing significant risks to both animal and human health. Subtherapeutic doses of antibiotics have traditionally been used to enhance growth and improve economic efficiency in poultry farming. However, these practices have facilitated the emergence of resistant microbial strains, threatening global health security and prompting a search for sustainable alternatives. This review highlights the significance of phytogenic as feed additives (PFAs) as promising substitutes for antibiotic as feed additives (AFAs) in poultry production. PFAs, derived from plant-based compounds, exhibit multiple beneficial properties, including antimicrobial, antioxidative, anti-inflammatory, and immune-modulatory effects. Moreover, they offer the potential to produce high-quality organic poultry products while reducing the likelihood of microbial resistance. Despite these advantages, inconsistent results among studies underscore the importance of standardized approaches to maximize their efficacy. This review aims to evaluate the current status of antibiotic use in poultry farming globally, explore the properties and mechanisms of PFAs, and assess their potential as viable alternatives to antibiotics. By consolidating available knowledge, this review provides insights into the benefits and challenges associated with PFAs, offering guidance for future research and practical applications in sustainable poultry production.

## INTRODUCTION

Using antimicrobials in farming systems enhance livestock productivity and contribute to meeting the global demand for animal protein sources. Antibiotics in poultry production aim to enhance growth performance and reduce production costs. Subtherapeutic doses of antibiotics as feed additives (AFAs) have been demonstrated to improve growth performance and overall farming economics [1, 2]. They are also used to treat various bacterial infections in poultry, such as those caused by *Salmonella* and *Escherichia coli*, *Listeria*, and *Clostridium*. [3]. Meanwhile, there is growing global concern about the increasing antimicrobial use trends projected to reach over 105,000 metric tons in food-producing animals by 2030 [4]. Worldwide uncontrolled antimicrobial use contributes to antimicrobial resistance and poses significant risks to animal and human health [5, 6]. Hence, despite the importance of AFAs in poultry nutrition and growth performance, their usage is increasingly monitored because of concerns that farmers often apply these drugs without oversight, veterinary recommendations, or suitable technical guidance [7].

A study has reported examples of microbial pathogen resistant strains first identified in livestock due to the use of AFAs and then transmitted to humans [8]. For example, the multidrug-resistant strain *Salmonella typhimurium*, identified in livestock, is the primary cause of Salmonellosis in humans [9]. The drug-resistant strain *Campylobacter jejuni*, which causes human infection, was also associated with antimicrobial use in poultry farms. According to a previous study by Akosile *et al*. [10], antibiotic cross-resistance can also develop in humans because antibiotics are chemically similar to antibiotics used in animals and poultry. The essential role of AFAs in livestock and poultry production may decline in the future due to emerging policies aimed at reducing their use.

Alternatives are needed to reduce antimicrobial reliance, and research has focused on developing antibiotic substitutes, such as phytogenic substances, which offer several advantages in poultry nutrition [11]. This review aims to comprehend the current status of AFA and consolidate the available knowledge and technical information about the potential properties, effects, and uses of phytogenic as potential alternative to AFAs in poultry production.

## THE CURRENT STATUS OF ANTIBIOTIC USE WORLDWIDE

It is reported that several studies have indicated that antimicrobial use in livestock is twice that of humans, but it has received little attention from experts and scientists worldwide. The concentration of antibiotics used in countries varied [12]. China was the largest antibiotic producer and consumer in the world in the field of livestock production before 2020 [13]. However, in 2018, Russia and in 2020, China issued regulations that restricted the use of certain veterinary antibiotics in livestock production. This includes banning the use of certain critically important antibiotics, such as fluoroquinolones and colistin [14]. This regulation could reduce antibiotic use in Russia and China in future strategies. According to the available reports, Russia, Brazil, China, India, and South Africa were the major (58%) antimicrobial producers and consumers in the world in 2020 and are anticipated to be at the top of the list and increase their use double by 2030 (Table 1) [5].

**Table 1:** Antimicrobial use trends: current and future predicted use by 2030.

No.	Region/Country	Global use of antimicrobials by 2020	Predicted increase in antimicrobial use by 2030
1	Global	99,502 tons (95% CI 68,535–198,052)	Will increase by 8.0% (107,472 tons [95% CI: 75,927–202,661])
2	Asia	58,377 tons, 59% of global demand	6%
3	South America		14%
4	Europe		5%
5	North America		4%
6	Africa		25%
7	China	32,776 tons (56%)	1%
8	Pakistan	2,184 tons	Increase by 44% (3,143 tons)
9	Brazil		13%
10	Australia		Will increase by 16%
11	Russia		13%
12	USA		3%
13	India		5%

https://journals.plos.org/globalpublichealth/article/figure?id=10.1371/journal.pgph. 0001305.g001, CI: Confidence interval

Poultry farms may be the focal point of antimicrobial use, particularly in developing countries. The increasing demand for poultry products (meat and eggs) has led to the risk of developing antibiotic-resistant microbes in developing countries [8]. Due to the development of antibiotic resistance in human pathogens, the use of AFAs has been banned for decades in various countries worldwide [13].

Swann’s committee in the United Kingdom in 1960 restricted or banned the use of oxy-tetracycline as AFAs because microbial resistance to this drug in humans was reported to be transferred from animals. Likewise, the European Union withdrew the use of AFAs in livestock production in 2006 [15]. This reduced actual antibiotic exposure. The government of the Netherlands set a target to reduce the use of AFAs by 70% by the end of 2015. Food processing companies such as McDonald’s and Tyson foods set strategies to source chicken from antimicrobial-free poultry farms. However, the ban on the use of antimicrobials as feed additives has resulted in increased production costs and animal welfare, and subsequently, a rise in the use of antibiotics for veterinary therapeutic purposes has been reported. This was a documented event in Denmark, along with a 30% reduction in antimicrobial-resistant strains in food-producing animals; subsequently, antibiotic use for therapeutic purposes has been increased by 33% by veterinary practitioners at the clinical level. On the other hand, with the increasing demand for poultry meat at a rate of about 14% and an increased 11% of human population globally [12], it has become crucial to develop appropriate and attainable alternatives to AFAs without compromising poultry productivity and welfare.

## ACTION MECHANISM OF AFAS

A study conducted by Suresh *et al*. [16] focused on the effects of antibiotics as growth promoters, but to date, no clear-cut action mechanism of antimicrobials as growth-promoting agents has been recognized. Preliminary theories have linked antimicrobial action to reduced microbiota [17], resulting in reduced competition for nutrient utilization [18], and lessened microbial metabolites that affect growth performance (amino acid and bile catabolism). This theory was challenged by a previous study reported by Niewold [19], who found that antibiotics interact with host immune cells and lower the inflammatory response, resulting in reduced levels of pro-inflammatory cytokines that lessen appetite and increase muscle catabolism. Niewold [19] stated that the anti-inflammatory role of antimicrobials improves energy harvest, leading to improved productivity. It has been reported that the gut microbiota increases the thickness of the gut lumen, leading to morphological changes, resulting in a reduction in nutrient absorption and triggering the host immune response due to their antigenic recognition [20, 21]. The literature revealed that AFAs exert their effect as growth promoters in poultry through two mutual mechanisms of action that is the “bacterial-centric” effect by inhibiting the normal gastrointestinal microbiota, which results in an increase in nutrient availability as well as a reduction in maintenance cost. Studies have also shown that decreased gastrointestinal microbiota leads to improved fat use as these bacteria degrade bile salts, resulting in decreased fat digestibility. The second mechanism of the growth-promoting effect of AFAs is “host-centric,” which can be attributed to their immune-modulatory effects on the host. It is understood that the AFAs have a “growth permitting” effect rather than “growth-promoting” effects [19].

Considering the mode of action of AFAs, the main characteristics of alternative to AFAs are their interaction with host immune cells, modulation of the inflammatory response, and targeting of sites differently from antibiotics as antimicrobial activity with different action mechanisms, thus preventing drug-resistant strains. The “host-centric” mechanism of action of purposive AFA alternatives reduces the risk of developing microbial resistance development [16]. However, the beneficial effects of many alternatives are well documented, whereas the effects of inconsistency from farm to farm are a concern for which the reason is still not well understood. Therefore, the mechanism of action that can evade antimicrobial resistance is important to understand [22]. Consequently, AFAs alternatives should potentially target host-centric rather than bacterial-centric approaches to avoid microbial resistance. Many researchers have recommended using AFA alternatives in combined formulas for better efficiency, as well as suggest further studies to be conducted for better recognition of the effects and mode of action [13].

## PHYTOGENIC PROPERTIES

Phytogenic plants or phytochemicals produced by plants during their growth may have no nutritional importance. These secondary metabolites can be used in feed as additives to improve animal productivity and health. The phytogenic, also called botanicals, possess antioxidative, antimicrobial, anti-inflammatory, and immune-modulatory properties [23]. Phytogenic categorizes the plant part as a herb that includes the plants’ herbs, leaves, and flowers (non-woody), and spice or non-leafy parts (seed, fruit, bark, and root) can be included in these categories. The main bioactive component of phytogenic feed additives (PFAs) is polyphenols, whose composition and concentration differ depending on the plant and plant parts (herbs and spices), geographical area, plant harvesting stage, environmental factors, and processing techniques [24, 25].

Phytogenic feed additives have been demonstrated to improve feed efficacy and livestock productivity by inducing morphological changes in the intestine that increase the size of the villi and crypt, resulting in an increase in the secretion of intestinal mucosa, saliva, and bile acid. It has also been reported that the incorporation of phytogenic substances as feed additives in poultry diets leads to high-quality, drug-free, and organic poultry products that are safe for human consumption [26]. Several studies have been conducted to evaluate the response of livestock and poultry to various PFAs and have demonstrated improved body weight gain, feed efficiency, and feed conversion ratio (FCR), while other study did not show significant effects [22]. Consistent with the meta-analysis report [27] prepared from 13 studies as well as the majority of the studies conducted in the past decade reviewed under this review, phytogenic use in broiler production has shown increased growth performance, feed efficiency, and other biochemical parameters [28] mitigate stress [29], improved antioxidant status [30], enhanced carcass quality characteristic [31], antimicrobial and immunomodulatory effects [32], and improved anti-inflammatory effects [1].

Similarly, medicinal plant leaf meal dietary supplementation is recommended as a commercial antibiotic replacement due to their similar effects on performance, carcass quality, and internal organ characteristics of antibiotics used in poultry production [33]. The positive effects of phytogenic materials are also mainly attributed to their antimicrobial [34] and antioxidant properties [29]. Bacterial resistance is a complex combination of various active molecules with different modes of antimicrobial activity [16]. Phytogenic plants also have immunomodulatory effects, such as enhanced immune cell proliferation, increased cytokine expression, and improved antibody titers [35].

The variation in the results of different studies reflects the tendency of phytogenic effects on poultry production. For example, Kim *et al*. [36] reported improved feed efficiency and reduced FCR in poultry fed with essential oil (EO) supplementation. In contrast, Jang *et al*. [37] reported no significant effect on the production performance of EO in poultry diets. Franz *et al*. [38] attributed the variation in the results to differences in the composition, type, origin, inclusion level, and environment of the EO used, as well as the conditions of the trials. The potential use of phytogenic depends on their properties, knowledge of their secondary components, mechanism of action, and safety of use for animals and humans [39]. Therefore, in order to have a consistent result, the entire affecting factors have to be standardized.

## ANTI-INFLAMMATORY EFFECT

Chronic stress associated with cytokine release, dysbiosis, and the so-called leaky gut syndrome caused by oxidative reactions leads to inflammation. This can primarily affect the functioning of gastrointestinal cells, leading to cell thickness, damaging the gastrointestinal barrier and nutrient utilization, and threatening poultry productivity. All types of stress may be biological, social, environmental, nutritional, physical, chemical, or psychological, leading to inflammatory reactions affecting the animal’s health and productivity [1]. Due to chronic inflammation, oxidative stress can rise, and reactive oxygen species (ROS) are produced, leading to cell membrane lipid, mitochondrial, and endo-membrane peroxidation that results in cell death [40]. Notably, 90% of all pathological disorders in poultry are associated with intestinal chronic inflammation [41]. Poultry gut health is directly linked to its microbiota balance, which can directly affect intestinal lining health, biological activities, and nutrient absorption, leading to impacts on systemic immunity, neuroendocrine activity, and productivity [6, 42]. Therefore, it is important to prevent oxidative stress and intestinal inflammation as the “secret killers,” especially in poultry farming.

Several synthetic substances are available that can significantly reduce inflammation, thereby lowering animal reactions and improving productivity. Natural substances containing anti-inflammatory phytochemicals have attracted the interest of nutritionists and are positively tested as feed additives in poultry production [30, 43]. These substances also improve poultry productivity, digestive enzymes, and nutrient use. Several phytochemical substances, such as polyphenols, triterpenes, flavonoids, terpenoids, alkaloids, and plant sterols, are known to have one or more of the aforementioned properties [44].

## ANTIOXIDANT EFFECT

Cells and tissues of living organisms are exposed to free radicals and other ROS reactions even during normal oxygen metabolism, which play a role as signaling molecules involved in maintaining homeostasis [45]. However, extreme stressors can elevate ROS levels, resulting in lipid peroxidation, damage to cell membranes and DNA, and modifications to small GTPases [46]. Consequently, these processes can contribute to the development of chronic stress symptoms. The phytogenic additives possess antioxidant properties that protect lipids from oxidation [10], increase cytokine levels, reduce oxidative stress, improve immune responses and pancreatic and intestinal enzyme levels, and have anti-inflammatory effects in the living body [35]. These effects lead to increase in antioxidant activity in various tissues, reductions in oxidative stress, and improved health and production [24].

Previous studies indicated that plant oils have natural antioxidant properties that can improve the oxidative stability of meat and meat products and retard the oxidative deterioration process [10, 29, 30]. This is possible when the antioxidant and oxidative stressor (free radicals) balance is maintained by supporting antioxidant enzyme activities through phytogenic-derived antioxidants [47]. The use of dietary phytochemicals such as carvacrol, thymol, catechins, quercetin, oregano, curcumin, and cinnamaldehyde is regarded as a sustainable strategy that can be adopted for improving the antioxidant capacity within the body (live animals) to alleviate oxidative stress as well as to enhance the growth performance and meat quality in poultry [48].

The phytogenic-containing diet has also been reported to considerably improve egg production and quality traits in a layer-hens-fed diet supplemented with 200 g/ton feed; this effect was attributed to the phytogenic antioxidant properties [49]. Phytogenic substances have the potential to reduce lipid peroxidation and improve the antioxidant components that protect the product against oxidative damage [50]. In a study, improved gut microbial balance and increased production performance were attributed to the antioxidant properties of phytogenic foods when the poultry diet was supplemented with 1% phytogenic substances [51].

It is reported that the antioxidant properties of polyphenols are well recognized in various *in vitro* and *in viv*o studies [52, 53]. Polyphenols, as natural hunters, play a role in scavenging free radicals and improving cell ability and the synthesis of ROS-removing enzymes. These components scavenge free radicals through various mechanisms of action, such as H-atom transfer from the OH group (s) of polyphenols to free radicals and single electron transfer to the free radicals [52]. The presence of hydroxyl (OH) groups is an active component of the antioxidant properties of polyphenolic that can donate hydrogen to free radicals generated during lipid oxidation. By the way, prevent or reduce the production of OH peroxide groups [26]. The antioxidant properties of phytogenic plants may also be attributed to their metal-chelating ability, hydrogen atom donation, scavenging of peroxide, superoxide anions, and nitric oxide-free radical capability [50].

Polyphenols scavenge free radicals through several mechanisms. First, they can transfer hydrogen atoms from their OH groups to free radicals. Second, they facilitate a single electron transfer to these free radicals [54]. Research indicates that polyphenols effectively eliminate various ROS and reactive nitrogen species, such as hydroxyl radicals (HO), peroxy radicals (ROO), superoxide anions (O_2_−), and peroxynitrite (ONOO−) through these mechanisms [55]. The next mechanism is the chelation of transition metal ions such as Fe_2_+ and Cu_2_+, which limits the production of HO groups. In copper or hydrogen peroxide systems, polyphenols exhibit prooxidant properties, further preventing the generation of HO [56].

**Figure 1:**
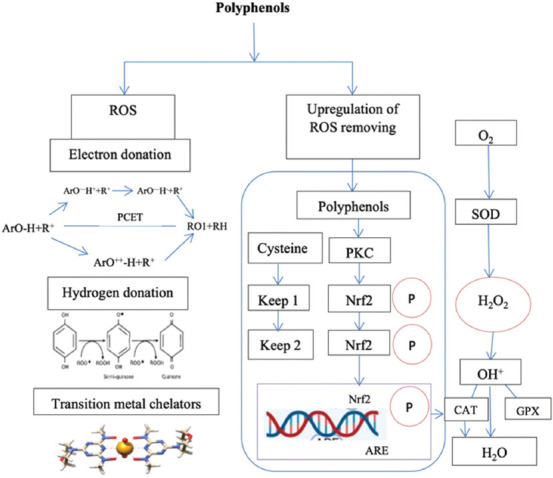
Mechanism of the antioxidant effects of polyphenols as natural antioxidants [1].

From this review, we found that previous studies have reported that phytogenic-sourced polyphenol compounds can reduce oxidative stress by improving antioxidant enzyme activities and altering signal transduction pathways [24, 35]. This results in cell protection and improves overall performance, productivity, and internal physiological conditions in poultry. Polyphenols inhibit pro-oxidant enzymes such as membrane-associated β-nicotinamide adenine dinucleotide oxidase, xanthine oxidase, and protein kinase C [57]. The antioxidant effect of polyphenols as natural antioxidants is illustrated in Figure 1 [1].

Polyphenols also play a role in alleviating NO-mediated oxidative stress [58] and preventing the oxidation of certain antioxidants, including ascorbate and tocopherols. The green tea compound epigallocatechin-3-gallate protects neurons against oxidative stress by activating the expression of heme oxygenase [58].

Various phytogenic substrates exert antioxidant and anti-inflammatory effects on broilers (Table 2) [10, 23, 29, 33, 35, 59–69].

**Table 2:** The anti-inflammatory and antioxidative effects of phytogenic substances.

Phytogenic substances	Bioactive component	Dose used	Potential effect	References
Turmeric rhizome extract	Curcumin	0.1–0.3 g/kg diet in broiler	• Decreased MDA • Enhanced antioxidant enzyme activity No effect on serum creatinine, total proteins, or liver enzyme levels	[10]
*Taraxacum mongolicum* aqueous extract	Taraxasterol, flavonoids, polysaccharides, polyphenols, chicoric acid, 1,3-dihydroxyacetone dimer, L-malic acid	1000 mg/kg broiler diet	Improved serum, liver, and ileum antioxidant capacity in broiler	[23]
Coriander seed	Linalool	0.5% in broiler diet	• Increased daily weight gain • Improved antioxidative activity of SOD, CAT, and total antioxidant capacity. Reduced MDA concentrations	[29]
Turmeric powder	Curcumin	0.3 or 0.6 g/kg broiler diet	• Neutralized superoxide radicals • Increased the activity of SOD and Chloramphenicol acetyltransferase (CAT) Decrease in malondialdehyde (MDA) levels in broilers	[35]
Cannabis seed	Flavonoids, terpenes, and cannabinoids	0.3% in poultry feed	• Improved intestinal health • Enhanced serum quality	[59]
*Quillaja saponaria* and *Yucca schidigera* (saponins)	Steroidal saponins, such as sarsasapogenin, smilagenin, and yamogenin	250 mg/kg diet in broiler	• Anticoccidial	[60]
*Ganoderma* triterpenoids	*Ganoderma* triterpenoids	10 mg/kg diet in broiler	• Reduced inflammation in chickens	[61]
Essential oils derived from basil, thyme, and sage	Eugenol, thymol, carnosol, and carnosic acid	0.05% inclusion in the broiler diet	• Improved production performance, antioxidant capacity, and concentration of polyunsaturated fatty acids in thigh meat • Improved intestinal *lactobacilli* • Decreased numbers of *Staphylococcus* spp. and *E. coli*	[62]
Essential oils from basil, thyme, and sage	Eugenol, thymol, carnosol, and carnosic acid	0.05% of the diet in broiler	• Improved antioxidant capacity • Improved production • Lowered *E. coli*, *coliforms*, and *Staphylococci* in the small intestine and cecum and an increased *lactobacilli* count	[62]
Grape seed extract	Proanthocyanidins and flavonoids	0, 0.01, and 0.02% in duck diet	• Improved duck growth • Increased serum superoxide dismutase (SOD), glutathione peroxidase, and total antioxidant activities	[63, 64]
Cinnamon bark essential oil	Cinnamaldehyde	300 mg/kg broiler diet	• Improved immunological response in broiler chicks • Reduced cecal counts of *E. coli* and *Clostridium* spp. • Increased SOD activity	[65]
Condensed tannins from grape seed extrac	Tannins	125, 250, 500, 1000, and 2000 mg/kg diet for 42 days in broiler	• No effects on growth performance and mortality are observed • Decreased MDA content, serum cholesterol, and low-density lipoprotein levels, increased the liver glutathione.	[66]
*Artemisia*	Artemisinin	1 and 1.25 g heat-stressed treated broiler	• Decreases in plasma corticosterone, alanine aminotransferase, and MDA and aspartate aminotransferase. • Increases in tri-iodothyronine/thyroxine, tri-iodothyronine, and SOD	[67]
Cinnamon bark essential oil	Cinnamaldehyde	300 mg/kg diet in broilers	• Improved immunological response • Low cecal *E. coli* and *Clostridium* spp. Counts, improved intestinal villi height, and increased serum SOD	[68]
*Phyllanthus niruri* Linn and *Melaleuca cajuput* (Kajuput and Meniran) leaf	Phyllanthin, niruriflavone terpenoids such as cineol, limonene, 4-terpineol, a-terpineol, a-pinene Linalool	1% and 2% of diet in broiler production	• No effect on body weight gain, feed intake, feed conversion ratio, average carcass and internal organ weight • Increased weight of the Fabricius bursa	[33, 69]

## ANTIMICROBIAL EFFECT

It is commonly acknowledged that phytogenic activities as possible substitutes for antibiotics can combat infections at the microbiological level [26]. It’s reported that numerous studies have documented the effectiveness of PFAs as antibacterial agents [70, 71]. Secondary metabolites, including phenolic acids, are principally implicated in the therapy of microbial infections, and the vast majority of these metabolites show strong bactericidal and bacteriostatic properties [72]. Bacteriological examinations showed that the *E. coli* colony-forming unit count was reduced in the fecal samples of laying hens supplemented with marigold (*Tagetes erecta*), dandelion (*Taraxacum officinale*), dried calendula (*Calendula officinalis*), and dried basil (*Ocimum basilicum* “Genovese”) leaves [73]. The phytogenic substance was suggested as a possible substitute for controlling *Clostridium perfringens* and necrotic intestinal infection and specific combinations of EO components to limit their colonization and growth in the gastrointestinal tract. It was further confirmed that PFAs prevent the proliferation and growth of *E. coli* and *C. perfringens* is expressed in the large and small intestines, respectively [74].

On the other hand, research has shown that supplementation with eugenol and trans-cinnamaldehyde downregulates the gene expression profile of *Salmonella* in the cells of the intestinal epithelium of broiler chickens [75]. In addition, adding phytogenics to broiler nutrition enhanced the levels of immunoglobulin G and immunoglobulin M levels [76]. These antibodies produce targeted immunity that hinders specific viruses and harmful bacteria from adhering to the gut wall [77]. Numerous pathogenic bacterial species, such as *Enterobacter cloacae, Listeria monocytogenes, Pseudomonas aeruginosa*, *E. coli*, *Pseudomonas fluorescens*, *Staphylococcus aureus*, and *Pseudomonas putida*, are adversely affected by carvacrol in terms of development and survival [72]. The diverse functional groups of phytogenic agents lead to variations in their antimicrobial mechanisms [78]. For instance, thymol and carvacrol, while both exhibiting antibacterial properties, act differently against Gram-negative and Gram-positive bacteria [70]. Essential oils such as thyme, oregano, and clove contain phenolic compounds such as eugenol, carvacrol, and thymol, which contribute to their antibacterial effects [11].

The OH group of phenolic terpenoids has antimicrobial properties. The intensity of action of microorganisms is determined by the existence of potential functional groups. This was further supported by the discovery that substances produced from plants have a low molecular weight and contain flavonoids, alkaloids, glucosides, polyphenolics, saponins, and EOs [78]. Thymol has well-analyzed active ingredients in EOs and contains the monoterpene 5-methyl-2-(1-methyleth-yl) phenol, which works on many Gram-positive and Gram-negative bacteria [70]. Bacterial and fungal growth was inhibited by the gas phase of clove and cinnamon oils. It is believed that EOs within bacteria block intracellular proteins and expel necessary cytoplasmic compounds, ultimately leading to bacterial death [70]. Spices and herbal products have been confirmed to act as antibacterial agents by altering cell membranes and triggering ion leakage, thereby reducing pathogen virulence [71]. Some phytogenic, such as flavonoids and catechins, disrupt cell membrane synthesis by binding to peptidoglycan layers, which inhibits ribosome and peptidoglycan production [79]. In addition, phytogenics can generate ROS in bacterial cells, leading to anatomical and physiological changes in membranes and causing damage through oxidative bursting at high concentrations [80].

Phytogenic feed additives can also reverse antimicrobial resistance, inhibit bacterial respiratory chain enzymes, and affect adenosine triphosphate synthase enzyme activity [81]. Thus, the antibacterial mechanisms of these ingredients in poultry nutrition involve multiple events that influence the entire bacterial cell [82]. The potential antimicrobial effects of the phytogenic agents are presented in Table 3 [32, 53, 62, 75, 83–87].

**Table 3:** Antibacterial effects of phytogenic substances.

Phytogenic substances	Bioactive component	Dose used	Potential effect	References
*Cannabis sativa* L. Leaves	Flavonoids, terpenoids, and cannabinoids	10 g/kg, 20 g/kg, and 30 g/kg in broiler diet	• Reduced fecal *Escherichia coli* counts	[32]
Basil, thyme, and sage are derived from essential oils.	Eugenol, thymol, carnosol, and carnosic acid	0.05% essential oil from each species	• Reduced number of *Staphylococcus* spp. and *Escherichia coli*	[62]
Trans-cinnamaldehyde and eugenol	Trans-cinnamaldehyde and eugenol	Low-dose=0.5% and 0.75%. High-dose=0.75% and 1% in broiler diets	• Reduced *Salmonella* Enteritidis colonization in broiler chickens • Downregulated expression of motility genes	[85]
Clove and cinnamon water extracts	Cinnamaldehyde and eugenol	500 mg/kg/day clove extract	• Inhibiting the growth of *Staphylococcus aureus* and *Escherichia coli*	[83]
Essential oils of thyme and anise	Oregano, carvacrol, yucca cinnamaldehyde	0.45 g/kg in broiler diet	• Reduced toxic effects of *Clostridium* *perfringens* in broiler chickens	[84]
Turmeric oleoresin Trans-cinnamaldehyde	Coated trans-cinnamaldehyde	500 mg/kg	• Reduced *Brucella intermedia* in the ceca	[85]
*Origanum vulgare* L. and *Origanum majorana* L.	Essential oil of oregano and marjoram	*In vitro* study of pathogens isolated from poultry meat	• Potentially inhibited the growth of *Staphylococcus aureus* in poultry meat.	[86]
Lemongrass and clove oil	Citral and eugenol	160, 80, 40, 20 and 10 mL/mL	• Significantly inhibited the growth of *Escherichia coli*, *Staphylococcus* aureus, and *Salmonella* spp.	[53]
Coriander essential oil and linalool	Petroselinic acid and linalool	1 and 4 mL/mL	• Biofilm formation by *Campylobacter* spp. • Inhibiting the growth of *Campylobacter jejuni* and *Campylobacter coli*.	[87]

## IMMUNE MODULATORY EFFECTS

Phytogenics have a significant immune-modulatory effect, enhancing overall animal health by regulating the immune system’s functioning. Phytogenics are bioactive compounds with immune-modulatory, anti-inflammatory, antioxidant, and antimicrobial properties [88]. Research has shown that phytogenics can improve gut health, feed palatability, and enhance gut development and performance through the use of bioactive compounds, such as polyphenols. These compounds exhibit antimicrobial, antiviral, antioxidant, anti-inflammatory, and immunomodulatory properties. The inclusion of phytogenic extracts in the diet of laying hens aged 19–74 weeks improved productivity, feed efficiency, and egg mass without compromising egg quality [89].

Phytogenics interact with the immune system by regulating cytokine production, activating immune cells, and enhancing antioxidant and anti-inflammatory activities. They can influence the production of pro-inflammatory and anti-inflammatory cytokines, improve the ability of the immune system to fight infection and reduce the risk of inflammatory diseases [1]. Phytogenics, which are rich in antioxidant and anti-inflammatory properties, enhance antibody production, phagocytic activity, and immune cell production in broiler diets, thereby reducing disease incidence. The primary function of PFA is to control potential pathogens and to beneficially modulate the intestinal microbiota [90]. Nuclear factor kappa B (NF-κB), a transcriptional factor, plays a crucial role in the inflammatory process, leading to the synthesis of proinflammatory proteins. Nuclear factor-erythroid 2-related factor-2 (Nrf2) is a redox-sensitive transcription factor involved in inflammatory processes. The Nrf2-antioxidant response element (ARE) pathway regulates the expression of antioxidant and detoxification enzymes and reduces susceptibility to inflammatory conditions. Previous studies by Huang and Lee [91] and Wang *et al*. [92] have reported that Toll-like receptors are vital to the innate immune system, triggering signaling pathways in poultry and livestock. Phytogenic compounds such as curcumin, caffeic acid, epicatechin, grape seed extract, cinnamaldehyde, and anthocyanins influence transcription factors to enhance oxidative stress defense, reduce inflammation, and promote animal health and growth performance [93].

Wang *et al*. [94] show that phytochemicals such as curcumin, caffeic acid, epicatechin, grape seed, and grape marc extract boost Nrf2 expression, reduce NF-κB activation, and protect against oxidative stress, inflammation, and coccidiosis infection in animals. Table 4 presents the immune modulatory effects of various phytogenic substances [95–115].

**Table 4:** Immune modulatory effects of phytogenic substances.

Phytogenic substances	Bioactive component	Dose used	Potential effect	References
A commercially available phytogenic feed additive (PFA)	-	150 mg/kg	• Improved hemagglutination inhibition titer in Newcastle disease • Reduced numbers of *Salmonella*, *E. coli*, and *Clostridium* • Improved body weight, feed conversion ratio, and retention of N and crude fiber	[95]
Hogweed (*Heracleum persicum*) Powder, flavophospholipol	• Furocoumarins • Polyphenols • Essential oils • Flavophospholipol	0.5, 1.0, and 1.5 kg/ton	• Increased plasma total protein and antibody titers for Newcastle disease virus.	[96]
Fermented *Ginkgo biloba* probiotics, and fermented *Camelia sinensis* probiotics	• Flavonoids • Terpenoids,	0.2% and 0.4%	• Enhanced immune system • Reduced pathogenicity of *E. coli*	[97]
Sage (*Salvia officinalis* L)	• Carnosic acid	0, 100, 200, 300, and 400 ppm	• Suppression of *E. Coli* in chickens • Improved antibody titers against virulent Newcastle disease • Enhanced eosinophil, monocyte, and heterophil counts	[98]
Fennel extract	• Volatile oils • Phenolic compounds	0, 100, 200, 300, and 400 ppm	• Enhanced immunoglobulin synthesis • Enhanced efficacy of Newcastle disease vaccination	[99]
Hogweed (*Heracleum persicum*)	• Furanocoumarins and Flavonoids	1.0, 1.5, 2.0, and 2.5 mL/L	• Increased titers of avian influenza and Lactobacillus microbial populations • Lowering *E. coli* ileum counts	[100, 101]
Thyme plant (*Thymus vulgaris*)	• Thymol • Essential oils • Phenolic compounds	50 and 100 ppm aqueous extract and 100 and 150 mg/kg feed of thyme powder, respectively	• Enhanced immunological response • Elevated influenza and Newcastle disease antibody titers	[102]
Chicory plant (*Cichorium intybus* L), pennyroyal (*Mentha pulegium* L.)	• Esquiterpene • Lactones and inulin • Coumarins	0.10%, 0.15%, and 0.20%	• Promoted the body’s resilience against Newcastle disease, growth of *lactobacilli*, and immune responses in the ileum and jejunum • Decreased *E. coli* counts and improved	[103]
Galbanum (*Ferula gummosa boiss*)	• Essential oils such as eucalyptus • Various terpenes and terpenoids	2.5, 5, and 10 g galvano/kg diet	• Stronger immunological response to Newcastle disease and defense against Gram-negative bacteria	[104]
Sumac (*Rhus coriaria* L.) and Sumac seed powder (SSP)	• Polyphenols, Tannins • Vitamin C	1, 2 or 3%	• Minimized *E. coli* numbers and enhanced health	[105, 106]
*Boswellia serrata* (BS)	• Boswellic acids • Terpenoids • Polysaccharides • Vitamin E	0.5, 1, and 1.5 g BS/kg diet	• Reduced total bacterial, fungal, and *E. coli* counts	[107, 108]
Green tea (*Camellia sinensis*)	• Polyphenols, Flavonoids • Amino acids, Vitamin C	0.25%–1.0% w/w	• Enhanced hemorrhagic reactions to influenza and Newcastle disease outbreaks • Decreased *E. coli* counts and increased *lactobacilli* counts in the broiler’s ileum and cecum	[109]
Turnip extract	• Gluconapin, Glucobrassicin • Sinigrin	0, 150, 300, or 450 ppm	• Antibacterial properties, such as reduced Gram-negative and coliform counts	[110]
Gave	• Hecogenin, tigogenin, and sarsasapogenin	500 ppm	• Increased titer of antibodies against avian influenza	[111]
Milk thistle (*Silybum marianum*)	• Silymarin compounds	0.5%	• Higher antioxidant capacity and defense against aflatoxin B1	[111]
Milkvetch (*Astragalus species*)	• *Astragalus* polysaccharides (APS) • Astragalus saponins • Flavonoids	0, 100, 200, and 300 mg/kg diet	• Increased antibody titers, as well as the levels of plasma interleukin (IL)-2 and IL-2 and interferon-) in chickens • Boosted immunity	[112, 113]
Arfaj (*Rhanterium epapposum*) and desert thorns (*Lycium shawii*)	• Flavonoids, Phenolic acids, Terpenoids	18 mg/kg diet	• Improved immunological response	[114]
Olive pomace	• Oleuropein • Hydroxytyrosol • Oleanolic acid	10%	• Increased antibody titers against infectious bronchitis and Gumboro disease	[115]

## CONCLUSION

This study comprehensively explores the potential of PFAs as sustainable alternatives to antibiotics in poultry production, addressing the critical challenge of antimicrobial resistance. It highlights the diverse effects of PFAs, including antioxidative, antimicrobial, anti-inflammatory, immune-modulating, and growth-promoting properties, while emphasizing both host as well as bacterialcentric mechanisms that target sites different from antimicrobials that evade microbial resistance risks. The strengths of this study lie in its comprehensive analysis, practical insights, and integration of biochemical and environmental factors influencing PFAs. However, variability in study outcomes and the lack of standardized protocols limit the generalizability of findings. Actionable recommendations include conducting systematic research to identify and standardize optimal PFA inclusion levels in poultry diets, developing guidelines for consistent processing, techniques, and application methods. Future research should also explore the applicability of PFAs across production sectors and focus on targeted education and outreach to ensure stakeholder engagement in adopting these sustainable practices.

## AUTHORS’ CONTRIBUTIONS

NA: Conceptualization and drafted the manuscript. AM, AZ, and OM: Literature review and manuscript writing. MNA: Literature review and manuscript writing. All authors have read and approved the final version of the manuscript.
